# Stroke in inflammatory bowel disease: a report of two cases and review of the literature

**DOI:** 10.1186/1477-9560-6-2

**Published:** 2008-03-21

**Authors:** Deepak Joshi, Tobias Dickel, Rakesh Aga, Gray Smith-Laing

**Affiliations:** 1 Department of Gastroenterology, Medway Maritime Hospital, Kent, UK

## Abstract

Thrombosis is a recognised complication of inflammatory bowel disease (IBD), in particular venous thrombosis. Arterial thrombosis, especially stroke is rare. There is a paucity of information regarding stroke in IBD and its management. The authors describe two cases of stroke in patients with IBD during periods of increased disease activity. The literature regarding this devastating complication and the procoagulant state that exists in IBD are reviewed.

## Background

Thrombosis is a well-recognised complication of inflammatory bowel disease (IBD) and is an important cause of morbidity and mortality. The commonest are deep vein thrombosis and pulmonary emboli [1]. Arterial thrombosis, in particular stroke, is rare in IBD. No guidelines are available at present to help manage this severe complication. The authors describe two cases of stroke in patients with ulcerative colitis (UC).

### Patient 1

**Figure 1:**
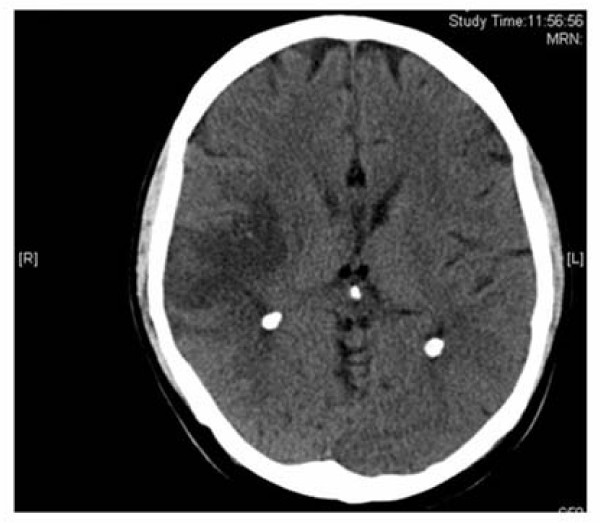
**CT demonstrating large ischaemic infarct in the right parietal lobe**.

A 55 year old, non-smoker, Caucasian man presented to the emergency room with left sided hemiparesis. The patient also had bloody diarrhoea (12 times a day). A diagnosis of ulcerative colitis had been made five years previously and the patient had been maintained on mesalazine only. The patient had been evaluated in the out-patient department three days earlier with an exacerbation of his UC and was started on prednisolone 40 mg daily only. There was no history of vascular disease. On examination the patient was normotensive (blood pressure 111/80 mmHg) and afebrile. Cardiovascular examination revealed normal heart sounds, and no carotid bruits. Abdominal examination revealed tenderness in the left iliac fossa and reduced bowel sounds. A dense left sided hemiplegia was noted. Blood tests showed a raised white cell count of 11.5 × 10^9^/L (NR 4–11) and C-reactive protein (CRP) of 29.6 mg/L (NR 0–5) and a thrombocytosis of 566 × 10^9^/L (NR 150–400). Fasting cholesterol of 3.3 mmol/L (NR 1.3–5.2) and blood glucose of 3.3 mmol/L (NR 3.3–5.8) were both normal. Stool cultures were negative. An electrocardiogram demonstrated normal sinus rhythm. No evidence of cardiomegaly was noted on a chest radiograph. Trans-thoracic echocardiogram and carotid dopplers were normal. Computed tomography (Figure 1) of the brain confirmed an ischaemic infarct in the right parietal lobe.

Flexible sigmoidoscopy demonstrated an inflamed granular mucosa with loss of the vascular pattern to the proximal sigmoid colon. He was commenced on intravenous hydrocortisone (100 mg QDS) and subcutaneous low molecular weight heparin (LMWH). Total parenteral nutrition was also commenced because of the likelihood of poor enteral absorption. After 14 days of treatment with hydrocortisone he underwent a total colectomy because of poor symptom control and worsening abdominal pain. Postoperative recovery was unremarkable. A thrombophilia screen (anti-thrombin, protein C and S, factor V leiden) was negative Twelve months post surgery the patient is mobilising independently with only mild weakness of the left upper limb.

### Patient 2

A 24 year old Caucasian woman presented to the emergency room with a left sided headache, confusion and increased bowel frequency (8 times per day). UC had been diagnosed six months earlier at colonoscopy and subsequently treated with mesalazine 800 mg BD and prednisolone 10 mg OD. On examination the patient was afebrile and normotensive (BP 119/80 mmHg). A global aphasia was noted with no other neurological deficits. Blood tests revealed a raised white cell count of 20.1 × 10^9^/L and CRP at 185 mg/L, and a thrombocytosis of 641 × 10^9^/L. Trans thoracic echocardiogram and carotid dopplers were normal. CT of the brain demonstrated an ischaemic infarct of the left middle cerebral artery territory. A thrombophilia screen was negative. The patient was managed conservatively and made an uneventful recovery with no residual neurological deficit but subsequently developed epilepsy. Her colitis has remained well controlled with azathioprine.

## Discussion

We describe two case of stroke in patients with known UC. Neither patient had any risk factors for cerebrovascular disease. One patient required a subtotal colectomy for failed medical treatment whilst the other patient made a full recovery and was spared surgery but developed epilepsy secondary to her stroke.

Deep vein thrombosis with or without pulmonary embolism remains the most common vascular event complicating inflammatory bowel disease. Thrombotic events involving the central nervous system are unusual. Cases of cerebral venous sinus thrombosis in IBD are well described [2,3]. Arterial thromboembolic complications occur less frequently [4] and the majority of cases seem to occur post surgery [5]. In a study of 7199 patients, only 7 patients with cerebrovascular disease were identified [6]. A review of the literature shows scattered case reports of arterial strokes in IBD [7-9]. Patients with ulcerative colitis appear to be more commonly affected than patients with Crohn's Disease [7]. Men and women are equally affected. Vitamin B6 deficiency leading to an acquired state of hyper-homocysteinaemia has also been described as a risk factor for stroke in IBD [7]. Active disease was also associated with the occurrence of stroke, but cases have been described during remission [4]. Pan-colonic disease has also been suggested as a risk factor for stroke. Significant morbidity is associated with arterial complications [10].

An increased risk of thrombosis is recognised in patients with IBD. The incidence of "thrombo-embolic (TE)" complications ranges between 1–8% [11]. The increased risk of TE complications appears to be unique to IBD when compared to other inflammatory conditions. Mieshler et al [12] demonstrated that IBD patients have a 3.6 fold higher risk of thromboembolism compared with controls. An increased risk was not seen in patients with coeliac disease or rheumatoid arthritis. The majority of TE cases (60%) in patients with IBD occurred during periods of increased disease activity or in the presence of gastrointestinal complications such as fistulae and abscesses [12].

All components of the coagulation cascade are thought to be involved in the pathogenesis of IBD [13]. This is the basis for the use of heparin as a treatment, and also accounts for the decreased incidence of IBD in patients with von Willenbrands disease and haemophilia [5]. A single cause for the hypercoaguable state that clearly exists in IBD has not been established. An increased frequency of Factor V Leiden, antithrombin deficiency and the prothrombin (G20210A) gene mutation have not been consistently identified [1]. Protein C and S levels have also been shown to be no different to controls [14]. Hyperhomocysteinaemia is associated with an increased risk of thrombosis. Homocysteine is generated via the metabolism of methionine and requires vitamins B6, B12 and folate. A point mutation (C677T) in the methyleneterahydrofolate reductase (MTHFR) gene can lead to increased serum levels. An increased incidence of the point mutation in the MTHFR in IBD patients with TE complications has not been established [1].

In the two cases described, both patients had evidence of active disease. Inflammation during episodes of increased disease activity is a potent prothrombotic stimulus due to fibrinolysis inhibition. Increased levels of pro-inflammatory cytokines (TNF and IL-6) and increased production of soluble CD40 ligand from activated platelets [15,16] are also thought to play an important role. Studies have also examined the role played by platelet-leukocyte aggregates (PLA) which are increased in patients with IBD, compared with healthy and inflammatory control subjects [17,18]. PLAs cause microinfarction and exacerbate thrombus formation by enhancing the production of tissue factor. Conventional risk factors for thrombosis such as immobility, dehydration, sepsis and surgery should not be forgotten.

Consequences of stroke in young patients can be devastating and even more complicated in the setting of active IBD. Conventional CT or magnetic resonance imaging should be performed to identify the anatomical distribution and the extent of the area affected. No guidelines are available for the treatment of stroke in IBD. The only evidence appears to be anecdotal, probably due to the paucity of cases. Treatment should involve the management of the underlying bowel disease and prevention or correction of acquired risk factors leading to the TE event. Novotony et al [10], proposed that all UC patients with an arterial TE event should undergo a colectomy due to the likelihood of pan-colonic disease and a poorer long term prognosis.

Prophylactic doses of LMWH are widely used in patients with IBD with increased disease activity, evidence of gastrointestinal complications, and during surgery or episodes of sepsis during hospital admissions. This is recommended by the British Society of Gastroenterology [19]. Anticoagulation, to prevent further thrombotic episodes, is a controversial because of the increased risk of intracerebral and GI bleeding, although patients have been successfully anticoagulated without bleeding complications [20]. Oral, colonic release, LMWH has now been used in the treatment of mild to moderate UC [21], and could represent a future therapeutic avenue. We suggest that all cases of stroke in IBD patients should be treated as in the general population with aspirin and modification of atherosclerotic risk factors. We do not advocate the need for total colectomy in the occurrence of an arterial TE event.

It is not uncommon for patients to present with de novo IBD with extra-intestinal manifestations which include vascular events. Clinical awareness of stroke as a complication of IBD is low. Guidelines are required to help guide physicians in the management of stroke in IBD. Investigations for thrombophilia should be reserved for those patients with atypical or recurrent thromboses or in those with a strong family history. Further research is needed to help establish the cause of the hypercoaguable state in IBD.

## Abbreviations

CT: Computed tomography; IBD: Inflammatory bowel disease; LMWH: Low molecular weight heparin; MTHFR: Methyleneterahydrofolate reductase; PLA: Platelet-leukocyte aggregates; TE: Thrombo-embolic; UC: Ulcerative colitis.

## Competing interests

The author(s) declare that they have no competing interests.
